# Approaching two decades of cystic fibrosis research in Qatar: a historical perspective and future directions

**DOI:** 10.1186/s40248-019-0193-4

**Published:** 2019-10-01

**Authors:** Samer Hammoudeh, Wessam Gadelhak, Atqah AbdulWahab, Mona Al-Langawi, Ibrahim A. Janahi

**Affiliations:** 1 Medical Research Center, Research Affairs, Hamad Medical Corporation, PO Box 3050, Doha, Qatar; 2 Pediatric Pulmonology, Pediatric Medicine, Sidra Medicine, PO Box 26999, Doha, Qatar; 3 Internal Medicine, Hamad Medical Corporation, PO Box 3050, Doha, Qatar

**Keywords:** Qatar, Cystic fibrosis, Cystic fibrosis transmembrane conductance regulator, CFTR I1234V mutation, Pancreatic sufficient

## Abstract

Cystic fibrosis (CF) is a genetic disease caused by a defect of CF transmembrane conductance regulator (CFTR) gene. CF affects multiple systems, predominantly with respiratory involvement. In Qatar, researchers have been exploring various aspects of the disease for almost 20 years. PubMed and Google Scholar were reviewed for articles related to CF in Qatar. The first publication appeared in the year 2000. Since then, several studies have been conducted on CF patients in Qatar considering a variety of topics. The presence of the CFTR I1234V mutation in a certain Arab tribe stands out as a distinguishing characteristic of CF patients in Qatar when compared to the larger Arab region or even worldwide. We aim here to summarize the existing CF research conducted in Qatar over the years as well as to introduce topics for future research.

## Background

Cystic fibrosis (CF) is a multisystem autosomal recessive disease caused by a mutation in the CF transmembrane conductance regulator (CFTR) gene [1]. Six classes of mutations currently exist, with the most common mutation being F508del, which is classified as a class II mutation [1]. Clinical manifestations include a range of symptoms involving the pulmonary, gastrointestinal, endocrine, and reproductive systems [2]. Morbidity and mortality related to CF are mainly due to respiratory disease [3] and gastrointestinal involvement [4].

CF has been diagnosed to date in a variety of ethnic and racial groups [5]; however, the global epidemiology of CF varies based on a number of factors involving health facilities, clinical awareness, registration systems, and research activities [6]. The prevalence of CF in Northern Europe is reported to be 1 in 2,500 to 3,000, with a carrier rate of 1 in 20 to 30 [5]. Separately, the prevalence of CF among non-Hispanic whites, Hispanics, African-Americans, and Asian-Americans is reported to be 1 in 3,200; 9,200, 15,000, and 31,000, respectively [5].

In Qatar, the total number of CF patients is 82, including 34 adults (18 males and 16 females) and 48 children (22 males and 26 females). In the pediatric population (*n* = 48), Qataris constitute 62.5% (*n* = 30) of cases, while non-Qataris constitute 37.5% (*n* = 18). The majority of these children 65% (*n* = 31) have the CFTR I1234V mutation, while 35% (*n* = 17) have other mutations [7]. Based on figures by the Planning and Statistics Authority in Qatar, the population of Qatar is 2,772,294 as of April 2019 [8]. The majority of the population is made of expatriates (88.4%), as Qatari’s represent the remaining 11.6% of the total population. The majority of the non-Qatari population comes from Asia: India 24%, Nepal 16%, Philippines 11%, Bangladesh 5%, and Sri Lanka 5%. The gender ration is highly skewed toward males (75%) which is due to a large influx of male laborers [9].

In the present study, PubMed and Google Scholar were reviewed for articles related to CF in Qatar, using the keywords “cystic fibrosis” AND “Qatar.” The references of identified articles were also searched for any additional relevant articles. This paper aims to briefly review the known research related to CF conducted in Qatar and to highlight possible future directions in order to attempt to bridge some of the gaps that currently exist in our knowledge, clinical practices, and patient care overall.

## The history of cystic fibrosis in Qatar

### The CFTR I1234V mutation

In a worldwide analysis, Bobadilla et al. reported that several CFTR mutations are commonly encountered globally; aside from F508del, the list includes G542X, N1303K, and G551D. As for the I1234V mutation, their analysis indicates that it is found in Saudi Arabia and France [10].

The I1234V mutation was first reported in the early 1990s in a sample from southern France [11]. Later on, El-Harith reported the presence of the I1234V mutation in two sisters from Saudi Arabia, who were both homozygous with symptoms of recurrent diarrhea and failure to thrive [12]. Banjar outlined the geographic distribution of mutations in Saudi Arabia and reported that the 1548delG and I1234V mutations are the top two most common CF mutations found among this population [13]. A report by the World Health Organization indicates that the I1234V mutation is rarely encountered globally, but is rather frequently encountered in the Middle East among Bedouin tribes [14].

In Qatar, Abdul Wahab et al. in 2001 were the first to report the presence of the homozygous I1234V mutation in patients from 17 families. The study showed that the families in question were from the same Bedouin tribe, with a 96.6% consanguinity rate, and had varying degrees of clinical manifestations [15]. More than a decade later, Molinski and colleagues examined the variant c.3700A > G in order to study its molecular consequences and found that it causes a splicing mutation rather than a missense mutation, which has initially been predicted to be caused by the variant [16]. More work related to patients in Qatar with the I1234V mutation will be highlighted in the next few sections.

Aside from the I1234V mutation, a few reports have been published to date discussing cases of non-Qataris with other mutations. Abdul Wahab et al. described the case of a Syrian CF child homozygous for the ΔF508 mutation with severe clinical manifestations [17]. The 1525-1G > A mutation associated with severe CF was found in a Pakistani child reported on by Abdul Wahab et al. [18]. The N1303K homozygous mutation was detected in an Egyptian child with pseudo-Bartter’s syndrome and was reported on in two case reports discussing the various findings [19, 20].

### Clinical features

The first publication about CF in Qatar appeared in the year 2000 when Abdul Wahab et al. reported on 45 patients with CF. Qatari children in this study had mild/moderate respiratory symptoms. More severe respiratory symptoms were reported among non-Arabic Asian cases, with an earlier occurrence of *Pseudomonas aeruginosa* colonization and pancreatic insufficiency as compared with in the aforementioned Qatari cases. The study showed that 26 out of 32 families came from the same Bedouin tribe, with consanguinity reaching a level of 98% [21].

Later on, Abdul Wahab described the characteristics of a Qatari adult female homozygous for the I1234V mutation. The patient was diagnosed late at the age of 35 years, was multiparous, and had symptoms of chronic lung disease [22]. Then, in 2006, exocrine pancreatic function, using fecal elastase-1, was measured in 40 patients homozygous for the I1234V mutation. This study showed that CF patients were characterized by pancreatic sufficiency. The study also found no difference between using polyclonal antibodies and using monoclonal antibodies when assessing exocrine function [23].

Sweat chloride concentrations [24] and growth parameters as well as calcium homeostasis [25] among CF patients with the I1234V mutation were investigated by researchers in 2009. Saadoon et al. studied nitric oxide levels among CF patients and found them to have lower fractional exhaled nitric oxide levels in comparison with controls [26]. Another study found a significant association between bone mineral density and forced expiratory volume in 1 s in a sample of 26 CF patients [27].

Later on, Bhat et al. compared imaging scoring systems as related to the results of pulmonary function testing [28]. Zahraldin and colleagues reported on two siblings with both CF and apparent mineralocorticoid excess and commented on the implications of such [29]. Abdul Wahab et al. studied zinc deficiency in CF patients and concluded that future research into the zinc levels of this patient population as related to *Pseudomonas aeruginosa* is warranted [30]. More recently, a study comparing adiponectin levels of 17 CF patients with the CFTR I1234V mutation to those of 18 healthy controls found that sputum adiponectin levels could be used to asses inflammatory status among CF patients in a minimally invasive manner [31].

### Microbiology

The study of the microbiology of CF patients in Qatar began in 2004 when Abdul Wahab et al. reported the case of a patient with *Achromobacter xylosoxidans* [32]. That same year, microbiological patterns of lower airway secretions were described in another paper. Researchers in this study isolated more than 100 pathogens from the samples of 36 CF patients. Typical findings included *Pseudomonas aeruginosa*, *Haemophilus influenza*, and *Staphylococcus aureus*. Less common pathogens were also found, including *Mycobacterium abscessus*, *Stenotrophomonas maltophilia*, and *Alcaligenes xylosoxidans* [33].

A few case reports were published during the following year. In the first, Janahi et al. discussed the possibility of a *Mycoplasma* infection in a female with CF and recommended future investigation of the long-term usage of macrolides [34]. In the second, Abdul Wahab et al. discussed the isolation of *Stenotrophomonas maltophilia* from a female CF patient with the I1234V mutation [35]. Antimicrobial resistance patterns in bacteria isolates were described by Elshafie et al. in a different paper published in 2007 [36].

Abdul Wahab later on studied the prevalence of *Candida dubliniensis* [37] and examined the genetic relatedness of *Pseudomonas aeruginosa* among CF patients and siblings with CF [38] as well as among CF versus non-CF patients [39]. During the following year, researchers compared the accuracy of bacterial identification methods using matrix-assisted laser desorption/ionization–time of flight mass spectrometry [40]. Then, in 2017, Abdul Wahab et al. investigated the presence of multidrug-resistant *Pseudomonas aeruginosa* among CF patients treated with inhaled antibiotics [41]. Separately, authors in another study examined lung function and body mass index in relation to the persistence of *Candida dubliniensis* [42].

### Other related work

Ben Omran and Abdul Wahab reviewed common genetic disorders in the Qatari population including CF in a book chapter, as part of a book discussing genetic disorders in the Arab world [43]. In 2015, Rehman et al. commented on an article published in *The New England Journal of Medicine* in regards to the usage of lumacaftor–ivacaftor among CF patients [44]. A few years later, Janahi et al. conducted a review on allergic bronchopulmonary aspergillosis among CF patients [45]. Additionally, Janahi and Rehman published two book chapters, discussing the clinical manifestations and management of CF in the first [46] and presenting a comprehensive review of the CF microbiome in the second [47]. Most recently, Al-Sadeq et al. conducted a review of CF mutations throughout the Arab world [48].

## Future directions and recommendations

### Screening programs

Vast strides have been made in an array of CF-related health care services as well as research activities in Qatar. The introduction of the National Premarital Genetic Screening Program for a number of diseases, including CF, represents one notable milestone that started in 2009. Other diseases screened based on high carrier frequency include: thalassemia, sickle cell disease, classical homocystinuria and spinal muscular dystrophy [49]. Despite the importance of such screening programs, a recent study showed low knowledge and attitudes towards these programs in the Qatari population. The study recommended using educational campaigns at the school and university level [50].

Currently in Qatar, Qatari patients are screened for the founder mutation c.3700 A > G (p.Ile1234Val). Negative cases are tested using the Elucigene CF29 mutation panel, which includes the core panel of 23 CF mutations that are recommended by the American College of Medical Genetics & Genomics. If no mutation was detected, a full CFTR exon sequencing is conducted.

Molecular carrier screening has been recommended by both the American College of Medical Genetics and Genomics and the American College of Obstetricians and Gynecologists in order for future parents to know their possible risk of conceiving a child with CF. [51] Newborn screening is another route for detection, which has now been implemented across the United States [51]. Moreover, molecular diagnostic testing allows for the earlier identification of cases, which in turn impacts the type of health care services received [51].

### Future research and potential therapeutic implications

The research by Molinski et al. provides invaluable insight into the disease for the local CF population, as related to the phenotype associated with a splicing mutation versus missense mutation, which in turn would reflect on the medical decision-making for these patients. The authors concluded that future treatment modalities are likely to be based on a better understanding of the molecular and clinical aspects related to the variations in CFTR mutations [16]. Building on these conclusions, it would be necessary to conduct genetic sequencing on the remaining CF patients in Qatar as well as their siblings and parents.

Moreover, further studies in the form of observational and experimental research are needed in Qatar. One of the possible future directions for clinical trials is triple-combination therapy, which has been approved by the United States Food and Drug Administration for those who are 12 years of age and older who have two copies of the *F508del* mutation or who have one mutation that is responsive to tezacaftor–ivacaftor [52, 53]. Research in other areas including the measurement of patient adherence to medication, dietary habits, and best lifestyle practices remains as topics to be untouched in relation to CF patients in Qatar; thus, intensifying health education efforts regarding these matters also represents another necessary approach.

In the future, establishing and maintaining a registry for CF patients in Qatar is crucial to building a reliable and comprehensive source of information for future research, public health, and health policy objectives [5, 54].

Gene editing using clustered regularly interspaced short palindromic repeats (CRISPR) technology is another research area that offers possible promising therapeutic implications, as altering DNA sequences allows for the repair of genetic defects [55]. Relating to this, several scientists have used the CRISPR technique to restore CFTR function, as shown in Schwank et al. [56], Crane et al. [57], Firth et al. [58], or Molinski et al. [59].

The use of organoids is another route for the development or enhancement of personalized medicine in the study of human organ systems [60, 61], as it enables the study of human diseases and development by modeling human organs using three-dimensional cultures to proceed [62, 63]. The performance of such research would be beneficial in a unique CF population with the CFTR I1234V mutation.

The role of dietary flavonoids is also an interesting arena for future CF research, as some scientists have suggested that flavonoids may have an impact on patients’ immune function and metabolism [64]. Others have hypothesized that flavonoids may have an anti-inflammatory role [65] or might activate the CFTR [66].

### Recommendations and conclusion

As for recommendations, establishing screening panels that are regionally and ethnically specific would help in increasing the detection rate of CF cases [10]. Furthermore, establishing incidence and prevalence statistics for CF-related disorders, locally and also regionally, would aid in future planning and help with determining the necessary size of health education efforts [14]. Finally, planning for the future care of CF patients requires the collaborative efforts of both national and international agencies and/or organizations [54].

In conclusion, the research conducted during the past two decades in Qatar has shed light on several aspects related to CF. However; further research work is necessary and encouraged in the upcoming decades in order to unravel the remaining mysteries associated with this debilitating disease.

## Data Availability

Not applicable.

## Abbreviations

CF: Cystic fibrosis
CFTR: Cystic fibrosis transmembrane conductance regulator
CRISPR: Clustered regularly interspaced short palindromic repeats
